# New Organic-Inorganic Hybrid Compounds Based on Sodium Peroxidomolybdates (VI) and Derivatives of Pyridine Acids: Structure Determination and Catalytic Properties

**DOI:** 10.3390/ma15175976

**Published:** 2022-08-29

**Authors:** Adrianna Sławińska, Malgorzata Tyszka-Czochara, Paweł Serda, Marcin Oszajca, Małgorzata Ruggiero-Mikołajczyk, Katarzyna Pamin, Bogna D. Napruszewska, Ewelina Prochownik, Wiesław Łasocha

**Affiliations:** 1 Jerzy Haber Institute of Catalysis and Surface Chemistry, Polish Academy of Sciences, Niezapominajek 8, 30-239 Krakow, Poland; 2 Faculty of Pharmacy, Jagiellonian University Medical College, Medyczna 9, 30-688 Krakow, Poland; 3 Faculty of Chemistry, Jagiellonian University, Gronostajowa 2, 30-387 Krakow, Poland

**Keywords:** peroxidomolybdates, X-ray powder data, crystal structure analysis, thermal decomposition, catalysis, Baeyer–Villiger oxidation, cytotoxicity

## Abstract

Two organic-inorganic hybrids based on sodium peroxidomolybdates(VI) and 3,5-dicarboxylic pyridine acid (**Na-35dcpa**) or N-oxide isonicotinic acid (**Na-isoO**) have been synthesized and characterized. All compounds contain inorganic parts: a pentagonal bipyramid with molybdenum center, and an organic part containing 3,5-dicarboxylic pyridine acid or N-oxide isonicotinic acid moieties. The type of organic part used in the synthesis influences the crystal structure of obtained compounds. This aspect can be interesting for crystal engineering. Crystal structures were determined using powder X-ray diffraction or single crystal diffraction for compounds **Na-35dcpa** and **Na-isoO**, respectively. Elemental analysis was used to check the purity of the obtained compounds, while X-ray Powder Diffraction (XRPD) vs. temp. was applied to verify their stability. Moreover, all the compounds were examined by Infrared (IR) spectroscopy. Their catalytic activity was tested in the Baeyer–Villiger (BV) oxidation of cyclohexanone to ε-caprolactone in the oxygen-aldehyde system. The highest catalytic activity in the BV oxidation was observed for **Na-35dcpa**. The compounds were also tested for biological activity on human normal cells (fibroblasts) and colon cancer cell lines (HT-29, LoVo, SW 620, HCT 116). All compounds were cytotoxic against tumor cells with metastatic characteristics, which makes them interesting and promising candidates for further investigations of specific anticancer mechanisms.

## 1. Introduction

Polyoxometalates (abbr. POMs) are a group of compounds with a great variety of remarkable structural and electronic properties which have found broad application in material science and catalysis. The variability of POMs arises from a wide range of types of crystal structures and also various properties. The increasing attention to these compounds is still observed. Typical for these compounds are metal-anion clusters MO_n_, linked by edges and corners. Metals are in the highest oxidation states (V^5+^, Nb^5+^, Ta^5+^, Mo^6+^ and W^6+^) [1,2]. Peroxido compounds can be considered as a part of the POMs group. Typical for such compounds are peroxo (O-O) bonds in clusters surrounding the transition metal. In mononuclear peroxido compounds, one can observe mono-, di-, tri or tetra- peroxidometalates, having 1, 2, 3 or 4 such bonds [3]. A detailed description of POMs and peroxo compounds was presented in papers by Conte and Floris [4].

Numerous POM structures revealed a variety of proprieties which give rise to a multitude of applications. POMs have found applications in catalysis, green chemistry and medicine. Cao et al. [5] presented a very thorough review containing different catalytic properties of POMs [5]. Wang et al. described electrocatalytic activity of Prussian blue analogue with POMs based hybrid compounds [6]. The activity of POMS in oxidation of cyclooctane, olefin epoxidation and epoxidation of alkanes is also known [7,8,9,10,11,12,13]. Application of POMs as a heterogenous catalyst was presented by Samaniyan et al. [14] and Pamin et al. [15]. An interesting review describing POMs in water solution as electrocatalysts, and their reactivity and stability, was presented by Gao et al. [16]. POMs are also known as active catalysts of the Baeyer–Villiger (BV) oxidation. In particular, Keggin type heteropolyacids and their salts were active catalysts in the BV oxidation with hydrogen peroxide [17,18,19]. Alternatively, transition metal hereropolysalts of Keggin type demonstrated high catalytic activity in the BV oxidation of cyclohexanone to ε-caprolactone in the oxygen-aldehyde system (Mukaiyama regime) [15,20], in comparison with appropriate heteropolyacids.

In addition, biological activity of POMs has been shown in many articles. POMs possess antiviral, antibacterial and antitumor activities, which was presented in a review paper by Authier [21]. The antibacterial properties and antitumor activity of the heteropolytungstates in aquaeous solution have also been reported by Ma et al. [22]. The anticancer properties of POMs were reported by Shin et al., Bijelic et al., Li et al., Hu et al., and Lu et al. [23,24,25,26,27,28]. Chaudhary et al. used polyoxo compounds as nano-inhibitors of amyloid aggregation [29]. A literature review article concerning interaction between POMs and biological molecules was presented by Rompuy et al. [30]. 

The results presented in this article are a continuation of our previous studies [10,11,12,31]. The synthesis procedure was similar, as previously described, and the inorganic core of the compounds was the same. The organic fragments were derivatives of pyridine acids: 3,5-dicarboxypyridine acid and N-oxide- isonicotinic acid. 

The goal of this article is presenting two new oxo-diperoxido-molybdenum complexes obtained with derivatives of pyridine acids. Their crystal structures and physicochemical properties, including–the infrared (IR) spectroscopy and thermal decomposition, are described. In addition, their catalytic properties in the Baeyer–Villiger oxidation of cyclohexanone to ε-caprolactone in the oxygen-aldehyde system are also presented. Three peroxide compounds of this family, which were obtained in our laboratory (two new ones, one obtained in 2017 [11]), were tested in the context of biological and, potentially, pharmacological activity.

## 2. Materials and Methods

### 2.1. Materials

Inorganic chemicals including sodium molybdenum oxide Na_2_MoO_4_ and NaCl were purchased from AVANTOR, Gliwice, Poland). 4-carboxylicpyridine acid (isonicotynic, **iso**), 3,5-dicarboxylicpyridine acid (**3,5-dcpa**) and H_2_O_2_ (30%) were purchased from Sigma–Aldrich Corporation (St. Louis, MO, USA).

### 2.2. Syntheses

The synthesis methodology similar to the procedure previously described by us was applied [10,11,12,31]. Both compounds were obtained by the reaction of reagents in cold 30% H_2_O_2_ solutions. Synthesis of **Na-35dcpa**, completed within 2 days, resulted in a solid sample in the form of powder. In the case of a second sample, **Na-isoO**, after 4 days of synthesis, single crystals were obtained. The yield of the reactions was greater than 50%. The purity of compounds was acceptable. 

**Sodium (µ-pyridine-N-oxo-3,5-carboxylato) (oxidodiperoxidomolybdate(VI)) trihydrate, (Na-35dcpa).**

0.005 mol NaCl and 0.005 mol Na_2_MoO_4_·2H_2_O were dissolved in 20 mL of cold H_2_O_2_ (30%). Then, 0.005 mol of 3,5-dicarboxylic pyridine acid was added to the resulting red solution. The unreacted 3,5-dicarboxylic pyridine acid was filtered off. The white powder (product) was obtained after 2 days in the filtrate solution. Yield 66% (1.09 g), C–19.24% (calc. 19.32%), H–2.019% (calc. 2.32%), N–3.28% (calc. 3.22%).

**Sodium [poly-bis(g2-peroxido)oxido-l-(1-oxo-jO-pyridine-4-carboxylate-jO)] molybdate (VI) hydrate, (Na-isoO).**

0.01 mol NaCl and 0.005 mol Na_2_MoO_4_·2H_2_O were dissolved in 20 mL of cold H_2_O_2_ (30%). The resulting red solution was acidified by drops of concentrated HCl until its colour has changed to yellow. Next, 0.005 mol of isonicotinic acid N-oxide was added, and the obtained yellow solution was stirred for one hour. Excess of undissolved isonicotinic acid N-oxide was filtered off. The 20 mL of 2-propanol were added to the filtrate and left to crystallize. Yellow crystals were obtained after 4 days; Yield 63% (0.986 g), C–20.76% (calc. 19.32%), H–2.145% (calc. 2.16%), and N–4.07% (calc.3.75%).

### 2.3. Methods

#### 2.3.1. X-ray Powder/Single Crystal Diffraction Data Analysis

The X-ray diffraction (abbr. XRD) investigations for **Na-35dcpa** and **Na-isoO** were performed at 293 K. The SuperNova diffractometer by Agilent Technologies (Santa Clara, CA, USA), currently Rigaku Oxford Diffraction, Frankfurt, Germany, (radiation CuKα, Sapphire2 detector, graphite monochromator), was used to collect the single crystal X-ray diffraction data. PANalytical X’Pert Pro MPD diffractometer by Malvern Panalytical, Almelo, The Netherlands (radiation CuKα, PIXcel 1-d detector) working in the Bragg–Brentano geometry was used to collect the X-ray powder diffraction data (abbr. XRPD). The preliminary analysis of phase purity was carried out with High Score software and the PDF-4+ database (ICDD, Newtown Square, PA, USA, 2019).

For the **Na-isoO** compound, crystal structure determination was based on a single crystal experiment. The single crystal structure solution by direct methods and refinement were achieved using SHELXS-97 and SHELXL-2013 programs, George M. Sheldrick, Göttingen, Germany [32]. To determine the location of hydrogen atoms, the difference Fourier maps of electron density were used. Anisotropic displacement parameters were refined for all non-hydrogen atoms.

For the **Na-35dcpa** compound, crystal structure determination was based on XRPD techniques. The sample was loaded into borosilicate capillary (2 r = 0.5 mm). A solution of the crystal structure was performed by direct space methods. Initial studies of powder diffraction data (indexing, space group determination, estimation of the reflection integrated intensities, structure solution by Direct Methods) were performed using the Expo2014 program [33]. Localization of heavy atoms (2 independent Mo atoms) was achieved using Expo2014 indicating good quality of the diffraction data and correctness of the unit cell. In structure solution procedure, positions of two Mo, two Na and two missing organic linkers (including details of their connection with inorganic cores) were determined using the direct (real) space methods (FOX program [34]). As mentioned previously, two independent molecules of 35-dcpa acid were used in the optimization process. The studied structure is very complex; it consists of two independent molecules in the asymmetric unit. The number of independent non-H atoms is 40. Missing water molecules, indicated by chemical analysis, were located using difference Fourier maps, calculated by JANA2006, Petříček, V., Dušek, M. & Palatinus, L. v.13/05/2019, Praha, Czech Republic [35].

To perform final refinement of the structure model, restrained Rietveld refinement was used (Jana 2006 [35]). Legendre polynomials were used to describe the background. To characterize the shape of diffraction maximum, a Pseudo–Voigt peak shape was used. Final Rietveld refinement plots are presented in Supplementary Material (Figure S1).

Table 1 shows the detailed crystal structure data for **Na-35dcpa** and **Na-isoO.** The Diamond [36] program was used for visualization of the crystal structures of the obtained compounds.

#### 2.3.2. IR Measurements

The IR spectra were recorded using a Bruker VERTEX 70 V (Bremen, Germany) spectrometer. The powdered samples were pressed into the pellets with KBr (1 mg sample for 100 mg KBr). The measurements were performed at room temperature. The Origin Pro v. 9.1, Northampton, MA, USA [37] program was used for the analysis of data.

#### 2.3.3. X-ray Thermal Decomposition

The thermal stability was tested using X’Pert Pro MPD diffractometer working in Bragg–Brentano geometry and equipped with a HTK-1200N high-temperature chamber manufactured by Anton-Paar. The X-ray data for **Na-35dcpa** and **Na-isoO** were recorded in the 2θ range from 5 to 55°. The measurement temperatures were: 25, 50, 75, 100, 125, 150, 175, 200, 225, 250, 300, 350, 400 °C and again 30 °C. The heating rate was 5 °C/min, and the cooling rate was 10 °C/min. 

#### 2.3.4. Thermogravimetry-Differential Scanning Calorimetry (TG/DSC)

TG/DSC measurements were carried out using a NETZSCH STA 409 Luxx instrument (NETZSCH-Gerätebau GmbH, Selb/Bavaria, Germany). The heating rate was 10 °C/min in the air atmosphere. The temperature range was settled from RT to 400 °C. The mass of the sample was 10 mg. The Origin Pro v. 9.1 [37] was used for the analysis of data.

#### 2.3.5. Brunauer, Emmett, Teller Methods (BET)

The BET surface area for peroxide compounds was measured with nitrogen adsorption at −196 °C on Quantachrome Autosorb-1, as described previously [31]. Before the experiment, each sample was degassed for 18 h at 25 °C under vacuum to remove adsorbed water and other surface impurities.

#### 2.3.6. Biological Studies

##### Cell Cultures 

All cell lines used in experiments were purchased from American Type Culture Collection (ATCC). Normal human fibroblasts BJ were used for experiments (ATCC designation CRL-2522; ATCC-LGC Standard, Teddington, UK), and the cells were cultured in Eagle’s Minimum Essential Medium, EMEM (Sigma-Aldrich, Seelze, Germany), supplemented with 10% foetal bovine serum (Eurex, Gdansk, Poland) and 1% antibiotic solution (penicillin-streptomycin, Gibco Laboratories, Grand Island, NY, USA). A panel of human colorectal cancer cell lines from ATCC was also used for experiments (HT-29, colorectal adenocarcinoma, HTB-38, SW 620, Duke’s type C colorectal adenocarcinoma, CCL-227, LoVo, Duke’s type C grade IV, colorectal adenocarcinoma). Tumor cell lines were maintained in L-glutamine containing Dulbecco’s Modified Eagle’s Medium (DMEM, Sigma-Aldrich, Seelze, Germany) with 4.5 g/L glucose, supplemented with 10% foetal bovine serum (Eurex, Gdansk, Poland) and 1% antibiotic (Gibco) [38]. The cell lines were passaged twice a week after previous treatment with trypsin (0.05%)/ethylenediamine tetraacetic acid (0.05% EDTA; Gibco) and grown in standard cell culture conditions at 37 °C in a humidified atmosphere of 5% CO_2_ in air as described elsewhere [39].

##### Cytotoxicity

The cytotoxicity of peroxidocompounds on cells was assessed by the 3-(4,5-dimethylthiazol-2-yl)-2,5-diphenyltetrazolium bromide (MTT) assay. The cells were cultured on a 96-well plate and treated with various concentrations of newly synthesized compounds at 1 μM/L, 10 μM/L, 100 μM/L, 1 mM/L, and 10 mM/L or Dulbecco’s PBS without Ca^+2^ and Mg^+2^ (a positive control) for 24 h. Then medium was replaced with a new one containing MTT reagent (50 μg/L), as described previously [40]. MTT formazan was solubilized in DMSO, and the absorbance was measured using a microplate reader Infinite M200 Pro (Tecan, Austria) at 550 nm (the reference wavelength was 690 nm). IC_50_ values were calculated from the concentration—response curves (IC_50_ was the concentration [μM/L] of a tested compound required to decrease the cell proliferation to 50% of the control) [41,42]. All data were expressed as arithmetic mean values with standard deviation (M; ±SD).

#### 2.3.7. Catalytic Activity in the Baeyer–Villiger Oxidation

The Baeyer–Villiger oxidation was performed as previously described [15,31]. The tests were carried out in a thermostated glass reactor at temperature of 40 °C, reaction time of 5 h and under the atmospheric pressure. In the typical experiment 0.01 mM of a catalyst, 4.6 mmol of cyclohexanone, and 14 mmol of benzaldehyde were dissolved in 10 mL of acetonitrile and stirred. The value of oxygen concentration in the reaction mixture was kept constant and controlled. Products of catalytic reaction (percent of consumed cyclohexanone and the percentage yield of ε-caprolactone) were analyzed by an Agilent 6890 N Gas Chromatograph (GC) equipped with an Innowax (30 m) column. During this analysis, the temperature was rising from 40 to 170 °C with step 20 °C/min. Before the proper GC analysis, the calibration process was performed for the standard reaction mixture. The results of cyclohexanone oxidation were calculated as the percentage yield in the presence of chlorobenzene as internal standard.

## 3. Results

### 3.1. Crystal Structure Data

**Figure 1:**
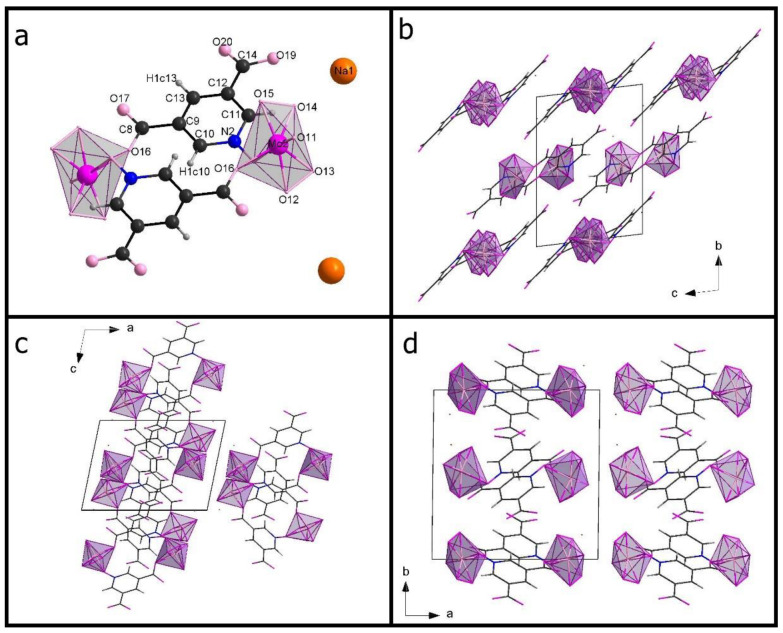
One of two centrosymmetric dimers in the asymmetric unit of **Na-35dcpa** (**a**); packing diagram of **Na-35dcpa** along the a-axis (**b**), along the b-axis (**c**), along the c-axis (**d**).

**Figure 2:**
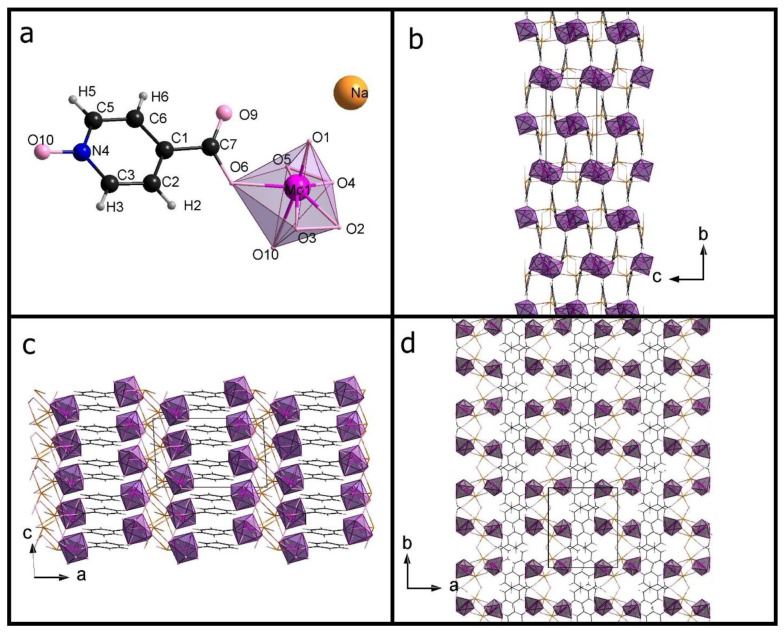
The asymmetric unit of **Na-isoO** (**a**); packing diagram of **Na-isoO** along the a-axis (**b**), along the b-axis (**c**), along the c-axis (**d**).

Table 1 shows the crystal data. Table S1 presents a list of selected bonds (Mo-O and O-O). Figure 1 presents an **Na-35dcpa** structure while **Na-isoO** is shown in Figure 2. Both compounds are sodium salts and inorganic-organic hybrids. The inorganic part contains a pentagonal bipyramid where the central, molybdenum atom is surrounded by oxygen atoms. There two peroxo groups and one apical terminal oxygen atom observed in each inorganic center. 

The first compound **Na-35dcpa** contains dimeric anions, where organic and inorganic groups are connected by two oxygen atoms. The linking oxygen atom belongs to carboxylic group C-O (1.28 (4) Å), O-Mo (2.06 (2) Å) and N-oxide group N-O (1.30 (2) Å), O-Mo (1.95 (1) Å). The central molybdenum atom is surrounded by seven oxygen atoms forming pentagonal bipyramid (MoO_7_). Two peroxo groups are observed in each MoO_7_ unit. The distance between molybdenum atom and one apical terminal oxygen atom (Mo=O) is 1.68 (2) Å. Moreover, the molybdenum atom is shifted from the equatorial plane of the pentagonal bipyramid by 0.403 (6) Å. The equatorial plane and the plane based on aromatic ring is twisted by 42.9 (5)° angle. In the structure, there are two independent anions and four in the unit cell (Z = 4). Both anions are built in the same way, so the description presented in this paragraph is valid for both of them (see Table S1).

The second compound **Na-isoO** contains MoO_7_ group—as inorganic core and isonicotinic acid N-oxide molecule—as organic ligand. The most interesting and important feature is a polymeric structure of the anion. Connection between molecules forming the chain is obtained by linking oxygen atoms, which belong to the carboxylic group C-O (1.305 (7) Å), O-Mo 2.067 (4) Å), and N-oxide group N-O (1.3341 (7) Å), O-Mo 2.304 (4) Å). Also in this structure, molybdenum atom is the central atom surrounded by seven oxygen atoms. Again, a pentagonal bipyramid is formed. One apical atom (Mo=O, 1.680 (4) Å) and two peroxo bonds can be found. The molybdenum atom is moved from the equatorial plane of the pentagonal bipyramid by 0.3347 (4) Å. The angle between the equatorial plane and the plane based on pyridine ring is 33.5 (2)°.

The organic parts were derivatives of pyridine acids, which were built in investigated structures. These were 3,5-dicarboxylicpyridine acid N-oxide or isonicotinic acid N-oxide, respectively. Interestingly, similarly to the syntheses with the use of 3,5-dcpa, H_2_O_2_ and potassium salts of Mo (VI), the formation of 3,5-dcpa acid N-oxide was observed [31]. 

The π–π interactions were observed between aromatic rings which can be expected for the pyridine derivative. The shortest distances between pyridine rings (measured as the distance between the centroid of one ring and the closest atom of the other ring) are, respectively, 3.22 Å for **Na-35dcpa** and 3.61 Å for **Na-isoO**.

### 3.2. IR Spectra

**Figure 3:**
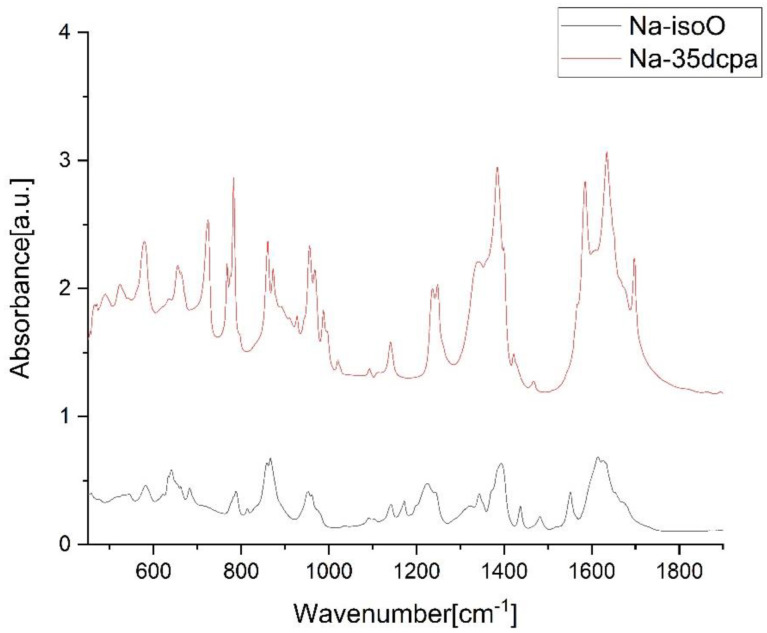
IR spectra of the compounds **Na-35dcpa** and **Na-isoO**. All range (400–4000 L/cm) wavenumber is presented in Figure S2.

The aim of IR examination was the confirmation of the presence of oxo- and peroxo- bonds and N-oxide vibrations. Table 2 and Figure 3 show detailed data for the studied compounds. Spectra for both compounds, as presented in Figure 3, are similar. Illustrated range of wavenumber representing inorganic part of compounds: (ν(Mo=O); ν_sym_(O-O); ν_sym_(Mo-(O)_2_); ν_asym_(Mo-(O)_2_)) and N-oxide vibrations. Peaks not classified in Table 2 represent organic parts of the compounds [3,9,10,11,12,31,43,44,45,46].

### 3.3. Thermal Decomposition—XRPD vs. Temperature

**Figure 4:**
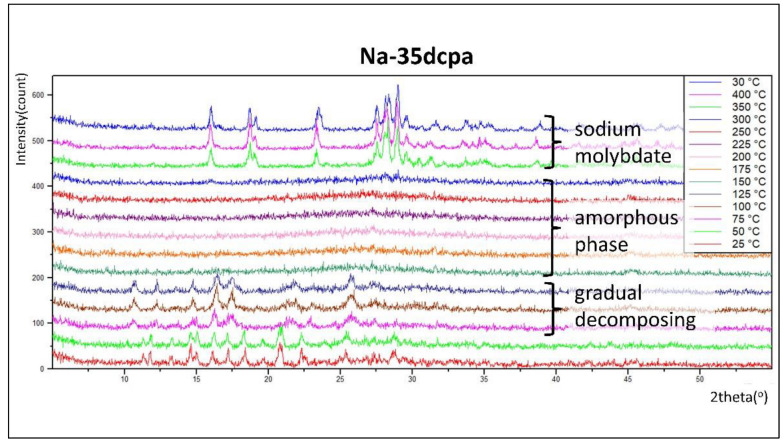
XRPD vs. temperature: thermal decomposition studies of the compound **Na-35dcpa** (see text for description). The estimated from Scherrer equation size of crystallites (initial phase 25 °C) was about ~75 nm (see Table S2 for details).

Relation between thermal stability and molecular structure was studied based on investigations of XRPD vs. temperature. The stability of **Na-35dcpa** and **Na-isoO** is unquestionable up to 75 °C and 50 °C, respectively. For the **Na-35dcpa** compound, gradual decomposing was observed in the range of 75–125 °C. Stepwise increase of the temperature results in the formation of an amorphous phase. After that, in the range of 350–400 °C, sodium molybdate was formed [PDF-4+ 04-017-3872]. This phase was stable after cooling to 30 °C (Figure 4). 

**Figure 5:**
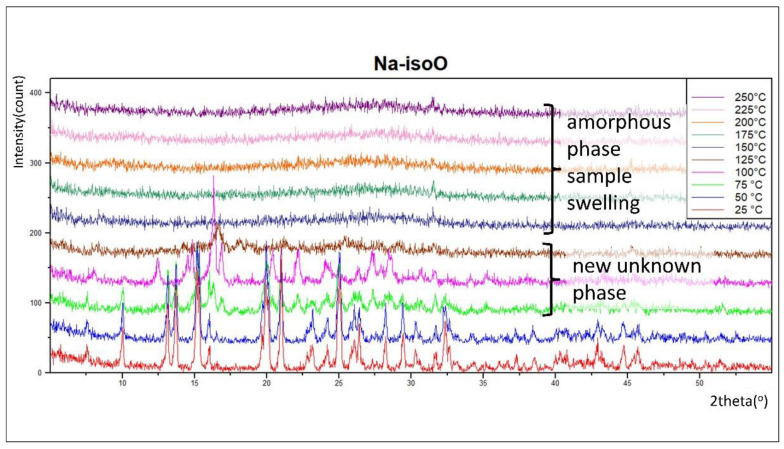
XRPD vs. temperature: thermal decomposition studies of the compound **Na-isoO** (see text for description). The estimated from Scherrer equation size of crystallites (at 25 °C) was about ~65 nm (see Table S3 for details).

For the **Na-isoO** compound (Figure 5), a new phase was observed in the range 75–125 °C. Unfortunately, the volume of the powder was changing during heating up to 100 °C and more (sample swelling). Measurements above 100 °C were affected by it and, as a result, XRPD patterns could not be interpreted (determination of lattice parameters and search in the ICDD pdf database failed). Above 125 °C, the sample is amorphous.

### 3.4. Thermal Decomposition-TG/DSC

The TG/DSC study correlates with X-ray thermal decomposition measurements. Slight temperature differences observed in these two methods result from a different step of the heating rates (TG/DSC gradual changes 10 °C/min.; the XRPD measurement-step 25 °C). 

Thermal decomposition of both samples is quite similar and can be divided into four stages depending on the weight loss. Calculated and observed mass losses qualitatively (due to complex and fuzzy thermal decomposition, especially for T > 350 °C) confirm it.

**Na-35dcpa** (Figure S3):

- In the range 75–150 °C, dehydration is observed (calc. weight loss 12%, obs. ~9%);
- At 185 °C, a sharp exothermic maximum due to the peroxo groups decomposition is visible (O_2_ emission, calc. 8%, obs. 7%);
- In the range 200–335 °C, decomposition of N-oxide and carboxylic groups occurs (2CO_2_ and ½O_2_ emission, calc. 24%);
- Above 325 °C, fast exothermic loss of the remaining organic fragments is observed;

**Na-isoO** (Figure S4):

- In the range of 75–175 °C, dehydration occurs (calc. 10%, obs. ~11%);
- At 265 °C, a sharp exothermic maximum related to the decomposition of peroxo groups is observed (O_2_ emission, calc. 10%, obs. ~9%);
- In the range 275–365 °C, decomposition of N-oxide and carboxylic groups takes place (CO_2_ and 1/2O_2_ emission, calc. 16%);
- Above 365 °C, fast exothermic loss of the remaining organic fragments is visible.

To conclude, it can be stated that, according to the expectations, the TG/DSC investigations suggest the higher stability of polymeric **Na-isoO**. The slow, lengthy heating process, typical of XRPD measurements, blurs these differences, making them invisible.

### 3.5. BET Analysis: Specific Area and Porosity Determination

The goal of BET measurements was to investigate the specific surface area (S_SA_), pore sizes and volume. The results are presented in Table 3. As described previously [31], all compounds show very low S_SA_, which originate from their crystal structure. For two compounds (**Na-35dcpa**, **Na-isoO**), the calculation of specific surface area was not possible because the S_SA_ was under 1 m^2^/g. Very low S_SA_ is characteristic for most of the POMs; however, it is not typical for potential catalysts for heterogeneous catalysis [43]. The pore sizes were different for each compound. The pore volume (BJH_des_ method) was observed to increase from 0.026 cm^3^/g to 0.068 cm^3^/g. The specific surface area was 2.048 m^2^/g for Na-nicO.

### 3.6. Biological Activity

**Figure 6:**
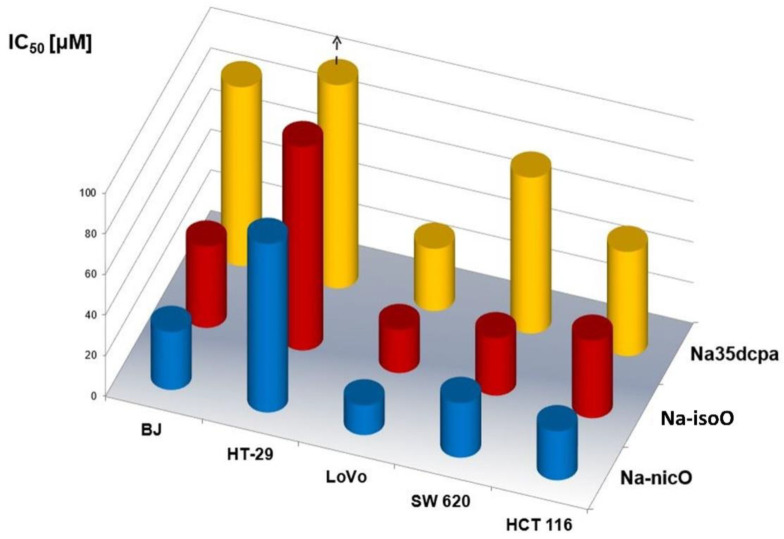
Comparative plot of IC50 values [μM] of **Na-isoO**, **Na-35dcpa** and Na-nicO against normal human fibroblasts (BJ) and human tumor cell lines (HT-29, LoVo, SW 620, HCT 116). Arrow up means higher value than presents on graph – see details on Table 4.

The emerging reports indicate that polyoxometalates demonstrate promising biological activities and the current state of knowledge in this field suggests that these inorganic metaloxygen clusters can be developed into a new generation of selective anticancer drugs [25] and may act against important human pathogenic bacteria [47]. In the present study, we employed the MTT assay to find out if newly synthesized peroxide compounds reveal biological activity on human cells. In particular, we aimed to find out whether the tested compounds may express any selectivity in cytotoxic action. The cytotoxic effects of **Na-isoO**, **Na-35dcpa** and Na-nicO against human cells are summarized in Table 4 with MT as IC_50_ values. Our study shows that Na-nicO was the most cytotoxic compound. However, all tested compounds were active against colon cancer cells (HT-29, LoVo, SW 620, HCT 116) and expressed considerable antiproliferative activity (Figure 6). At the same time, **Na-isoO** and **Na-35dcpa** were less active against normal human fibroblasts than against tumor cells and the molecular background of this finding will be further elucidated.

The newly synthesized peroxide compounds **Na-isoO**, **Na-35dcpa** and Na-nicO presented here possess significant biological activity. Such findings are consistent with the results regarding inhibitory activity of oxodiperoxido compounds previously reported by our team [31]. It should be noted that three compounds tested in the present study possess the same stabilization sodium cation and the same inorganic part–molybdenum atom coordinated by seven oxygen atoms—like the compounds synthesized previously. However, the compounds tested here differ in their organic part: **Na-35dpa** possesses 3,5-dicarboxypyridine acid N-oxide part, while **Na-isoO** contains 4-carboxypyridine acid and Na-nicO–3-carboxypyridine acid N-oxide. Moreover, **Na-isoO** is obtained as a polymeric structure, while Na-nicO and **Na-35dcpa** are obtained as dimers. In addition, **Na-35dcpa** possesses two dicarboxyl groups in contrast to two other compounds which have only one carboxyl group in each organic ligand. All differences presented here between organic parts of the studied compounds might influence their biological activity, but no unequivocal trend was found. Bijelic et al. [25] extensively discussed antitumor activity of different types of polyoxometalates as well as a specific role of the organic part of hybrid compounds, and presented a similar conclusion in their review [25]. However, it should be noted that the sodium atom is usually used as a stability cation of POMo or POV structures [25,48,49]. Probably, the resulting antiproliferative effect may come from a synergy generated by both the cationic species and the type of organic part of compounds, and the latter probably acts as an indispensable component of the cytotoxic activity of the molecule. Therefore, we may speculate that **the inorganic part of the compounds tested here containing bipyramidal pentagonal molybdenum center with seven oxygen atoms was necessary to excite the observed antitumor effect.** Considering the intracellular mechanism behind the inhibitory effect of hybrid compounds, some authors suggest that cytotoxicity is related to the disruption of the cell membrane and, in some cases, the interference with cellular metabolism leading to apoptosis may also be involved [25]. Further detailed experiments are required to understand the exact mode of action of the tested compounds inside cells. However, the data presented here are in compliance with literature reports and constitute a prerequisite for further optimization of the molecular framework of peroxide compounds for potential use in pharmacological medicine.

Recent studies reported that polyoxometalates have prominent antiproliferative effect in many types of human tumor cells, such as breast cancer cells and hepatic tumor cells [48]. Interestingly, in the present study, the inhibitory effect of tested compounds on cancer cells also depended on characteristics of particular tumor cell lines, since all compounds effectively inhibited proliferation of metastatic cancer cells LoVo, SW 620 and HCT 116 while the antiproliferative effect on cell line HT-29 deriving from the primary tumor was the lowest (Table 4). This suggests that newly synthesized peroxido compounds may express specific activity against metastatic colon cancer cells, and more advanced study is needed to assess precise intracellular targets and mechanisms of such activity of the compounds.

### 3.7. Catalytic Activity in the Baeyer–Villiger Oxidation

**Figure 7:**
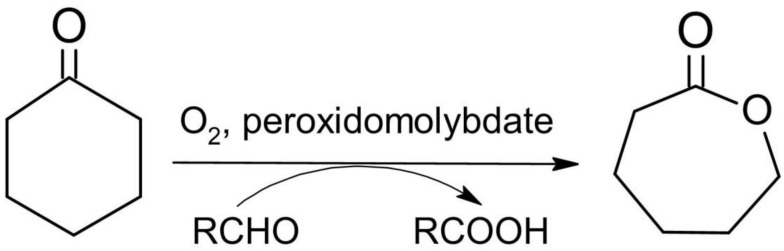
Scheme of the catalytics of the BV cyclohexanone oxidation with molecular oxygen and peroxidomolybdates as catalysts [31].

Two newly obtained compounds (**Na-35dcpa**, **Na-isoO**) and one synthesized by our group previously (Na-nicO) were used in the BV oxidation of cyclohexanone. Table 5 presents the results of the BV oxidation in accordance with the proposed reaction scheme (Figure 7).

Sodium oxodiperoxomolybdates used as catalysts in the oxidation of cyclohexanone to ε-caprolactone in the oxygen-aldehyde system demonstrate relatively high catalytic activity in the studied reaction. A comparison of newly synthesized samples with their potassium counterparts reveals that K-35dcpa shows the highest catalytic activity in the studied reaction. On the contrary, K-isoO is the least active among the studied catalysts. Finally, both Na-nicO and K-nicO are medium active catalysts demonstrating quite similar catalytic activity. Catalytic activities of sodium derivatives in the BV oxidation can be presented in the following series:

**Na-isoO** > **Na-35dcpa** > Na-nicO.

Comparing sodium and potassium compounds with the same organic part, we claim that:

- **Na-35dcpa** is weaker as a catalyst than K-35dcpa;
- **Na-isoO** is definitely a much better catalyst than K-isoO;
- Na-nicO and K-nicO have similar catalytic activity.

Presented results suggest that sodium oxidodiperoxidomolybdenum compounds having one carboxylic group in the organic part of the structure show higher or similar catalytic activity in comparison to their potassium counterparts in the BV oxidation. On the other hand, the sodium-derived compound (**Na-35dcpa**) containing two dicarboxylic groups in the structure exhibits lower catalytic activity than its potassium equivalent.

Considering chemically similar catalysts and similar reaction conditions (p, T), the results presented in our study can be compared with the results obtained for hetero polyacids of the Keggin type (based on Mo and W) [15]. Our catalysts turn out to be much better, they usually show higher conversion and definitely higher selectivity.

## 4. Conclusions

Two new oxidodiperoxidomolybdenum compounds were obtained and described. When using 3,5-dicarboxypyridine acid, a dimeric structure can be obtained, while using monocarboxylic isonicotinic N-oxide acid results in the formation of polymeric structure. Both compounds were stable at the room temperature and slightly above up to ~60 °C (XRPD vers. temp., TG/DSC). In addition, both compounds have very low specific surface areas which are below 1 m^2^/g and small pore volumes (**Na-35dcpa** 0.068 cm^3^/g, **Na-isoO** 0.028 cm^3^/g (value detected by BJH_des_) as well. 

Three examined compounds: **Na-35dcpa**, **Na-isoO**, and Na-nicO possess antitumor activity. All tested oxidoperoxido compounds may express specific activity against colon cancer cells. In addition, these compounds effectively inhibited proliferation of metastatic cancer cells LoVo, SW 620 and HCT 116. The obtained results are very promising and additional more advanced investigations are needed to find and learn more about mechanisms of such activity of these compounds.

Investigated compounds were found to be active in the BV cyclohexanone oxidation. For all sodium oxidodiperoxido compounds tested in the BV reaction, very high TON values were determined. Summarizing, the catalytic activity of investigated compounds is dependent on the type of alkali metal cation and on the type of pyridine derivative as well as the amount and position of carboxylic groups. In particular, sodium compounds with one carboxylic group in the crystal structure demonstrate higher catalytic activity than their potassium counterparts. An opposite effect was observed for sodium and a potassium compound with two carboxylic groups in the crystal structure. Obviously, the presence of the molybdenum part which forms a pentagonal bipyramide structure (molybdenum surrounded by seven oxygen atom) is essential.

## Figures and Tables

**Table 1:** Summary of crystal data of the investigated compounds.

Compound Code, (XRD Technique)	Na-35dcpa, Powder	Na-isoO, Single Crystal (s.c.)
Chemical formula	C_7_ H_10_ Mo N Na O_13_	C_6_ H_8_ Mo N Na O_10_
Chemical formula, structural	Na[MoO(O_2_)_2_C_5_H_3_NO(COO) (COOH)]·3H_2_O	Na[MoO(O_2_)_2_C_6_H_4_NO (COO)]·2H_2_O
MW (g/mol)	435.09	373.06
*T*(K)	293	−173
Wavelength λ [Å]	CuKα: 1.54187	CuKα: 1.54184
Crystal system, SG	triclinic, P-1	monoclinic, P2(1)/c
Cell parameters:		
*a* [Å]	12.2307 (7)	11.6753 (2)
*b* [Å]	12.2653 (7)	13.4514 (3)
*c* [Å]	8.9485 (6)	7.2919 (2)
*α* [°]	97.167 (4)	90.0
*β* [°]	101.840 (3)	93.602 (2)
*γ* [°]	87.893 (5)	90.0
V(Å^3^)	1303.48 (14)	1142.92 (4)
Z, calculated density (g/cm^3^)	4, 2.21	4, 2.156
Absorption coefficient (mm^−1^)	9.079	10.323
F(000)	740	728
Theta range [°]	5.007–79.992	3.793–80.579
Limiting indices	−10≤h≤9; −10≤k≤10; 0≤l≤7	−14≤h≤14; −17≤k≤16; −8≤l≤9
completeness to theta	100% (powder sample)	80.579, 98.2%
Absorption correction	Capillary, calc. for cylindrical sample	Multi-scan
refinement method	Rietveld	F^2^ (Fsqd)
Data/restraints/parame-ters	5712/83/86	2462/0/176
goodness of fit (on F^2^ in s.c.)	4.45	1.113
Final R indices (I > 2σ(I) in s.c.)	Rp = 0.0434, Rwp = 0.0632	R1 = 0.0575, wR2 = 0.1306
R indices (all data)	R_F_ = 0.0644	R1 = 0.0526, wR2 = 0.1281
Largest difference peak and hole (eA^−3^)	0.57, −0.47	0.775, −1.479
CCDC	2170626	2171243

**Table 2:** IR spectra vibrations and band assignments connected with oxodiperoxido- molybdate [3,9,10,11,12,31,43,44,45,46]. Vs—very strong, s—strong, m—medium, w—weak.

Compound	ν (Mo=O)	ν_sym_ (O-O)	ν_asym_ (Mo-(O)_2_)	ν_sym_ (Mo-(O)_2_)	(N-Oxide) Vibrations
**Na-35dcpa**	956 vs, 969 s	861 vs	580 s	523 m	493 w, 797 w
**Na-isoO**	953 s	867 vs, 586 vs	582 s	544 m	814 w

**Table 3:** N_2_ physisorption-derived parameters characterizing the obtained samples (S_SA_—specific surface area).

Compound	S_SA_ (m^2^/g)	Pore Size BJH_des_ (Å)	Pore Volume BJH_des_ (cm^3^/g)
**Na-35dcpa**	≤1	33, 62, 260	0.026
**Na-isoO**	≤1	53, 157	0.068
Na-nicO	2.48	33, 62, 115	0.028

**Table 4:** Cytotoxic effects (IC_50_, μM/L) as mean values with estimated SD of **Na-isoO, Na-35dcpa**, Na-nicO against normal human fibroblasts (BJ) and human tumor cells (HT-29, LoVo, SW 620, HCT 116). Experiments were repeated three times with similar results.

Compound	Normal Cells	Human Colon Tumor Cells			
		*Primary Tumor Cells*	*Tumor Cells Derived from Metastatic Sites*		
	Fibroblasts	HT-29	LoVo	SW 620	HCT 116
**Na-isoO**	40 ± 4.9	105 ± 9.1	21.1 ± 4.3	28.2 ± 1.0	38.1 ± 9.6
**Na-35dcpa**	87.9 ± 0	131.2 ± 6.9	30.6 ± 0.8	76.7 ± 1.7	51.3 ± 8.7
*Na-nicO*	28.5 ± 0.4	82.9 ± 11.8	14.6 ± 2.0	26.9 ± 3.7	24.1 ± 2.7

**Table 5:** The BV oxidation of cyclohexanone using molybdenum complexes.

Catalyst Number and Code	Conversion [%]	Yield [%]	Selectivity [%]	TON
**Na-35dcpa**	45.4	41.5	91.4	190.9
**Na-isoO**	50.0	48.8	97.6	224.5
Na-nicO	49.1	32.3	65.8	148.58
Selected examples from previous studies [31]				
K-35dcpa	90.9	89.7	98.7	412.62
K-isoO	37.6	6.8	18.1	31.28
K-nicO	44.7	35.6	79.6	163.76

## Data Availability

Crystal structure data have been deposited at the Cambridge Crystallographic Data Centre and allocated the deposition numbers CCDC 2170626 {**Na-35dcpa**}, 2171243 {**Na-isoO**} These data can be obtained free of charge via http://www.ccdc.cam.ac.uk/conts/retrieving.html (accessed on 22 December 2020), or from the Cambridge Crystallographic Data Centre, 12 Union Road, Cambridge CB2 1EZ, UK; fax: +44-1223-336-033; or e-mail: deposit@ccdc.cam.ac.uk. Supplementary data associated with this article can be found, in the online version, at https://www.mdpi.com/article/10.3390/ma15175976/s1.

## Supplementary Materials

The following supporting information can be downloaded at: https://www.mdpi.com/article/10.3390/ma15175976/s1, Microsoft Word v. 2013/2016 Figure S1: A: Na35-dcpa-Final Rietveld refinement plots [JANA2006 program]. The gray bar indicates the so-called ‘excluded region’—excluded due to the presence of diffraction lines from the ‘capillary optical system. B: Inset presents an enlarged high-angle range of the diffraction pattern. Figure S2: IR spectra results for **Na-35dcpa** and **Na-isoO**. Figure S3: TG/DSC results for **Na-35dcpa**. Figure S4: TG/DSC results for **Na-isoO**. Table S1: Interatomic distances for **Na-35dcpa** and **Na-isoO**. Table S2: Calculation average size of crystalline d(nm) for **Na-35dcpa**. Table S3: Calculation average size of crystalline d(nm) for **Na-isoO**.
