# Supplementary material for: New Organic-Inorganic Hybrid Compounds Based on Sodium Peroxidomolybdates (VI) and Derivatives of Pyridine Acids: Structure Determination and Catalytic Properties
Source: Materials (Basel). 2022 Aug 29;15(17):5976. doi: 10.3390/ma15175976 (PMC9457328; doi:10.3390/ma15175976)
Supplement: Supplementary file 1 [file materials-15-05976-s001.zip › materials-1869561-supplementary.pdf]

# Supplementary materials

# New organic-inorganic hybrid compounds based on sodium peroxidomolybdates(VI)and derivates of pyridine acids: Structure Determination and Catalytic Properties.

Adrianna Sławińska[1], M.Tyszka-Czochara[2], Paweł Serda[3], Marcin Oszajca[3], Małgorzata Ruggiero-Mikołajczyk[1], Katarzyna Pamin[1], Wiesław Łasocha[1,3,*]

[1]Jerzy Haber Institute of Catalysis and Surface Chemistry, Polish Academy of Sciences, Niezapominajek 8, 30-239 Krakow, Poland;

[2] Jagiellonian University Medical College, Faculty of Pharmacy, Medyczna 9, 30-688 Krakow, Poland

[3] Faculty of Chemistry, Jagiellonian University, Gronostajowa 2, 30-387 Krakow, Poland

Register

1. Selected lengths of bonds for **Na-35dcpa** and **Na-isoO**.
2. Rietveld refinement plots for **Na-35dcpa**.
3. IR spectra for **Na-isoO** and **Na-35dcpa**.
4. TG/DSC for **Na-isoO** and **Na-35dcpa.**
5. Calculation of average size of crystallites based on Scherrer equation

## 1. Selected lengths of bonds for **Na-35dcpa** and **Na-isoO.**

*Table S 1* Interatomic distances for **Na-35dcpa** and **Na-isoO.**

| **Na-35dcpa** | | **Na-isoO** | |
|---|---|---|---|
| atom1 – atom2 (symmetry code *) | | atom1 – atom2 (symmetry code *) | |
| Mo1-O1 | 1.68(2) | Mo1-O1 | 1.680(4) |
| Mo1 - O5 | 1.94(1) | Mo1-O4 | 1.929(5) |
| Mo1 – O2 | 1.94(2) | Mo1-O2 | 1.944(4) |
| Mo1 – O8(1-x,2-y,1-z) | 1.95(1) | Mo1- O3 | 1.959(5) |
| Mo1 – O3 | 1.95(2) | Mo1-O5 | 1.962(5) |
| Mo1 – O4 | 1.96(2) | Mo1-O6 | 2.067(4) |
| Mo1 – O6 | 2.08(2) | Mo1-O10 (1-x,0.5+y,0.5-z) | 2.304(4) |
| N1– O8 | 1.30(2) | N4-O10 | 1.334(7) |
| O4 –O5 | 1.47(3) | O4-O5 | 1.477(6) |
| O3 –O2 | 1.48(4) | O2-O3 | 1.480(6) |
| Second dimer ** | | | |
| Mo2 – O11 | 1.66(2) | | |
| Mo2 – O12 | 1.95(1) | | |
| Mo2 – O13 | 1.94(2) | | |
| Mo2 – O14 | 1.95(2) | | |
| Mo2 – O16 | 1.99(2) | | |
| Mo2 – O18(1-x,1-y,-z) | 2.47(2) | | |
| O12 – O13 | 1.47(3) | | |
| O14 – O15 | 1.47(3) | | |

**-if different than xyz in asymmetric unit*

***-see main text for explanation*

## **2.** Rietveld refinement plots for **Na-35dcpa**.

**(A)**

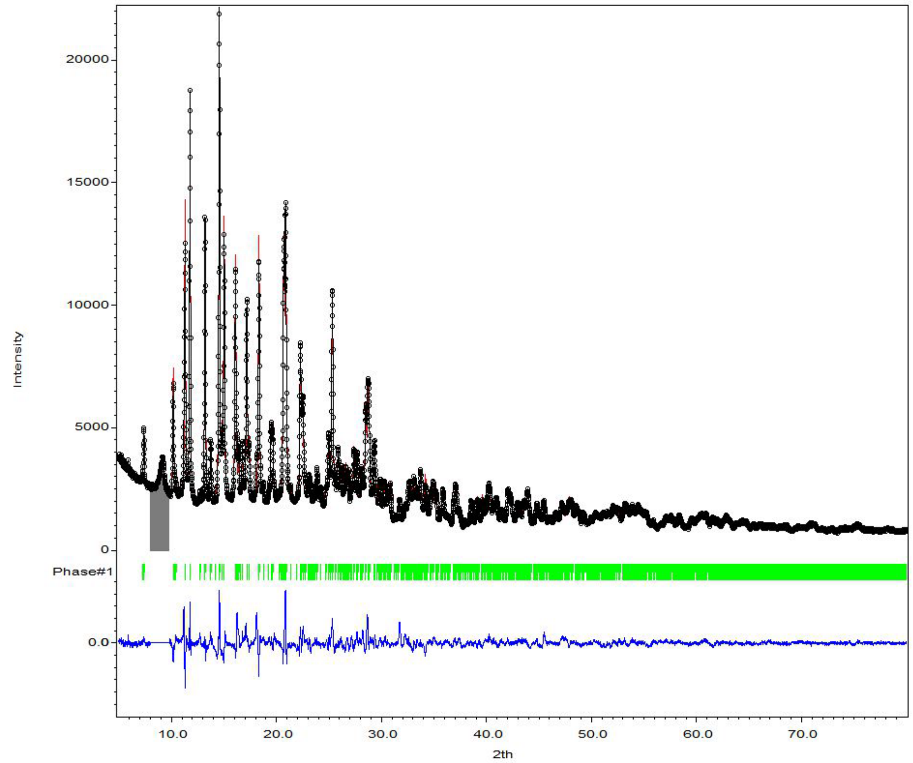


**(B)**

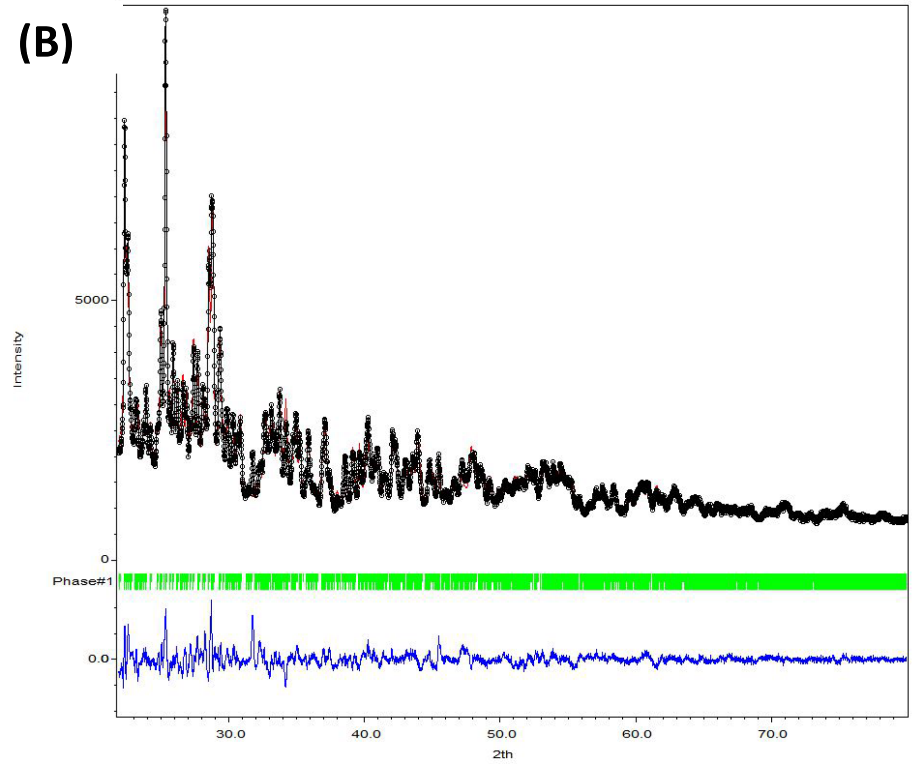


Figure S 1 (A): Na35-dcpa - Final Rietveld refinement plots [JANA2006 program]. Gray bar indicates the so-called 'excluded region' – excluded due to the presence of diffraction lines from the 'capillary optical system. (B): presents an enlarged high-angle range of the diffraction pattern. (Remark, Phase #1; indicate diffraction maxima from phase #1 -> Na-35dcpa).

## 3. IR spectra for **Na-isoO** and **Na-35dcpa**

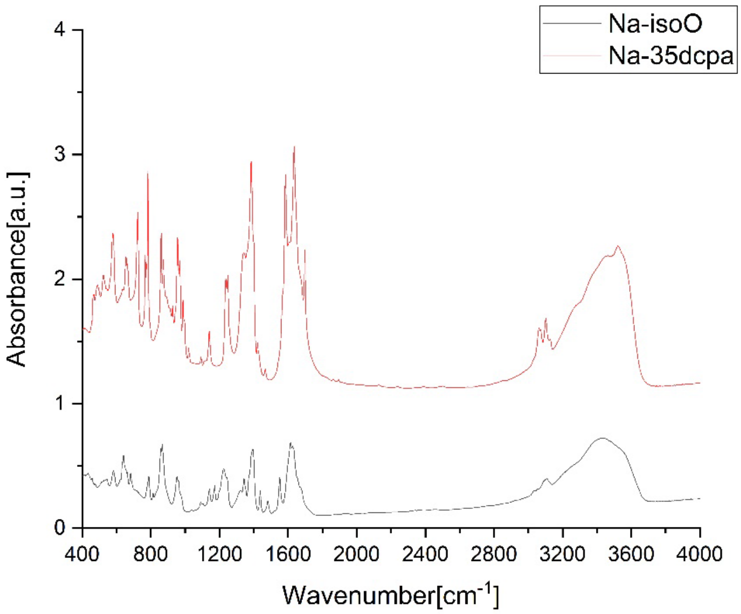


*Figure S 2 IR spectra for Na-35dcpa and Na-isoO.*

## 4. TG/DSC for **Na-isoO** and **Na-35dcpa**

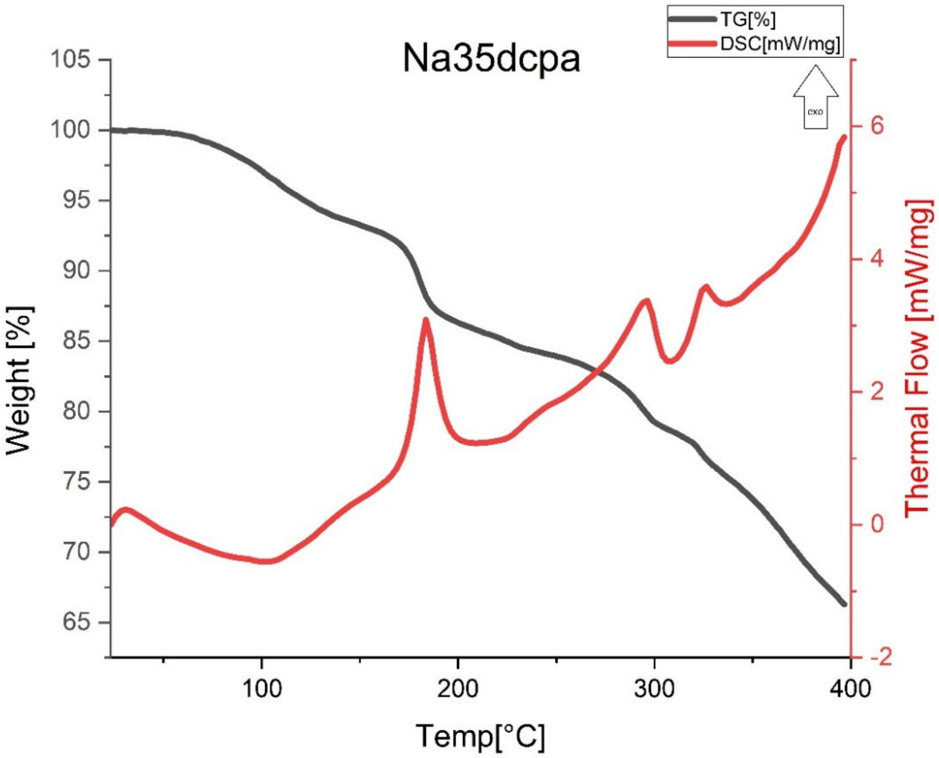


Figure S 3 TG/DSC results for **Na-35dcpa**.

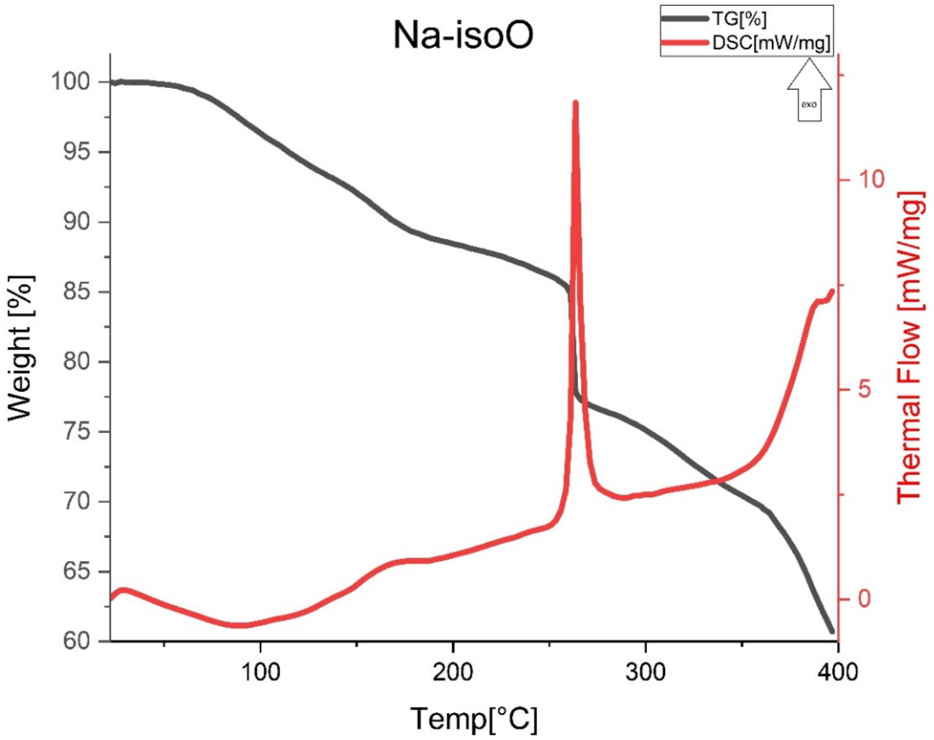


Figure S 4 TG/DSC results for Na-isoO.

## 5. Calculation of average size of crystallites based on Scherrer equation

Table S 2 Calculations of average size of crystallites d(nm) for Na-35dcpa.

| Compound | type of crystaliti | position(2θ°) | FWHM(2θ°) | d(nm) |
|---|---|---|---|---|
| **Na-35dcpa** | sfere κ=1 | 7.39 | 0.101 | 82.36 |
| | | 10.18 | 0.104 | 80.13 |
| | | 11.31 | 0.121 | 68.94 |
| | | 11,78 | 0.099 | 84.29 |
| | | 13.21 | 0.107 | 78.10 |
| | | 13.74 | 0.116 | 72.08 |
| | | 14.56 | 0.106 | 78.95 |
| | | 15.02 | 0.111 | 75.43 |
| | | 16.09 | 0.117 | 71.65 |
| | | 17.19 | 0.122 | 68.81 |
| | | 18.34 | 0.138 | 60.93 |
| | | **average (nm)** | | **75 (7)** |

Table S 3 Calculations of average size of crystallites d(nm) for Na-isoO.

| compound | type of crystaliti | position(2θ°) | FWHM(2θ°) | d(nm) |
|---|---|---|---|---|
| **Na-isoO**, | sfere κ=1 | 7.53 | 0.114 | 72.97 |
| | | 13.10 | 0.140 | 59.68 |
| | | 13.68 | 0.128 | 65.32 |
| | | 15.13 | 0.137 | 61.12 |
| | | 15.99 | 0.109 | 76.90 |
| | | 20.99 | 0.140 | 60.30 |
| | | 22.77 | 0.097 | 87.30 |
| | | 23.16 | 0.150 | 56.49 |
| | | 24.16 | 0.151 | 56.22 |
| | | 26.37 | 0.134 | 63.62 |
| | | 31.70 | 0.142 | 60.77 |
| | | **Avarage (nm)** | | **65 (10)** |

## 6. Summary

Summary of this passage. The materials tested: **Na-35dpca** and **Na-isoO** are made up of 'rather large' nanocrystals. They were investigated with the classical methods of X-ray powder diffraction. We believe that nanocrystals are large enough and have little influence on the specific surface area of the tested materials.
